# Association between physical activity and health-related quality of life: time to deterioration model analysis in lung adenocarcinoma

**DOI:** 10.1007/s11764-022-01259-z

**Published:** 2022-10-04

**Authors:** Jinman Zhuang, Yuhang Liu, Xinying Xu, Yuxin Cai, Maolin Liu, Zishan Chen, Shuyan Yang, Jianbo Lin, Zhijian Hu, Mingqiang Kang, Mengxin Lin, Fei He

**Affiliations:** 1 Department of Epidemiology and Health Statistics, Fujian Provincial Key Laboratory of Environment Factors and Cancer, School of Public Health, Fujian Medical University, 350122 Fuzhou Fujian Province, China; 2 Department of Health Toxicology, School of Public Health, Xiamen University, Xiamen, China; 3 Department of Thoracic Surgery, The First Affiliated Hospital of Fujian Medical University, Fuzhou, China; 4 Fujian Provincial Key Laboratory of Tumor Microbiology, Fujian Medical University, Fuzhou, China; 5 Fujian Digital Tumor Data Research Center, Fuzhou, China; 6 Department of Thoracic Surgery, Fujian Medical University Union Hospital, Fuzhou, China; 7 Department of Oncology, Fujian Medical University Union Hospital, Fujian Province, 350000 China; 8 Department of Pulmonary and Critical Care Medicine, Respirology Medicine Centre of Fujian Province, The Second Affiliated Hospital of Fujian Medical University, Quanzhou, China

**Keywords:** Lung adenocarcinoma, Physical activity, Health-related quality of life, Time to deterioration

## Abstract

**Background and purpose:**

Health-related quality of life (HRQoL) is a key aspect of care for cancer survivors that can be improved by physical activity. Our aim was to explore the relationship between physical activity and time to deterioration (TTD) of the HRQoL in patients with lung adenocarcinoma (LUAD).

**Methods:**

We conducted a hospital-based prospective study. The International Physical Activity Questionnaire long-form (IPAQ-L) was used to investigate the pre-treatment physical activity levels, and the EORTC Quality of Life Questionnaire version 3.0 (EORTC QLQ-C30) and EORTC Quality of Life Questionnaire-Lung Cancer (EORTC QLQ-LC13) were used to assess HRQoL at baseline and during follow-up. The QoLR package was used to calculate the HRQoL scores and determine TTD events (minimal clinically important difference=5 points). The effect of physical activity on the HRQoL was assessed using Cox regression analysis.

**Results:**

For EORTC QLQ-C30, TTD events of physical functioning (PF) and dyspnea (DY) in functional scales and symptom scales were the most common during follow-up. Pre-treatment physical activity was found to significantly delay TTD of insomnia (*HR*=0.635, 95%*CI*: 0.437–0.922, *P*=0.017) and diarrhea (*HR*=0.475, 95%*CI*: 0.291–0.774, *P*=0.003). For EORTC QLQ-LC13 scales, deterioration of dyspnea (LC-DY) was the most common event. Physical activity was found to delay the TTD of dyspnea (*HR*=0.654, 95%*CI*: 0.474–0.903, *P*=0.010), sore mouth (*HR*=0.457, 95%*CI*: 0.244–0.856, *P*=0.015), and dysphagia (*HR*=0.315, 95%*CI*: 0.172–0.580, *P*<0.001).

**Conclusions:**

Pre-treatment physical activity of LUAD patients may delay the TTD of multiple HRQoL indicators in EORTC QLQ-C30 and EORTC QLQ-LC13.

**Implication for Cancer Survivors:**

Health-related quality of life (HRQoL) is a key aspect of care for cancer survivors (someone who is living with or beyond cancer), that can be improved by physical activity. Our aim was to explore the relationship between physical activity and time to deterioration (TTD) of the HRQoL in patients with lung adenocarcinoma (LUAD).

**Supplementary Information:**

The online version contains supplementary material available at 10.1007/s11764-022-01259-z.

## Introduction 

Lung cancer is the leading cause of cancer-related deaths worldwide [1]. Lung adenocarcinoma (LUAD) accounts for approximately 40% of all primary lung tumors and is characterized by high mortality and metastasis rates [2]. Currently, surgery, adjuvant chemoradiotherapy, and immune checkpoint blockade therapy are the available curative options for lung cancer [3, 4], but the recurrence rate is still high (1-year recurrence rate of about 5%) [5]. In a recent report, the 5-year survival for advanced NSCLC was approximately 25% [6], an increase from the previous rate of 18% [7]. With an increase in the survival time, many lung cancer survivors experience health impairment [8]. Therefore, the improvement of the health-related symptoms of patients with LUAD is of much clinical relevance.

Health-related quality of life (HRQoL) is a broad multidimensional concept that includes perceptions of both physical and mental health [9]. It is a valuable index reflecting cancer survivorship outcomes [10]. Patients diagnosed with lung cancer were reported to experience a significant decline in psychosocial and physical function during and after treatment [11, 12]. Therefore, maintaining an adequate HRQoL is one of the goals of treatment for LUAD patients. Many factors can affect the HRQoL of patients with LUAD, and most attention is paid to modifiable behavioral factors [13]. Physical activity is a modifiable factor that is related to the prognosis of chronic diseases and cancer [14–16]. Previous studies have found that exercise prior to treatment or during rehabilitation can help improve outcomes of surgery, including cancer-related fatigue and dyspnea [17–19].

Physical activity is increasingly recognized as a valuable intervention as part of LUAD therapy. However, intermittent and missing HRQoL data in the follow-up period is a shortcoming of previous studies. The time to deterioration (TTD) model is a longitudinal time-event analysis used to assess post-treatment changes in the HRQoL of cancer patients over time, and it can address missing HRQoL data in long-term follow-up [20–22]. In this prospective study, we aimed to analyze the association between pre-treatment physical activity and the TTD in the HRQoL of LUAD survivors.

## Materials and methods

### Study patients

This was a hospital-based prospective study conducted in two hospitals in Fujian province (Thoracic Surgery and Respiratory Medicine of the First Affiliated Hospital of Fujian Medical University, Affiliated Union Hospital of Fujian Medical University). Patients recruited were newly diagnosed with primary LUAD, confirmed by fiber-optic bronchoscopy or histological examination, between May 2017 and November 2020. The inclusion criteria were (a) diagnosis of primary LUAD with pathological results and (b) patients able to answer the questionnaire clearly and autonomously sign an informed consent. The exclusion criteria were (a) patients diagnosed with benign lesions or secondary lung cancer; (b) patients lacking a pathological diagnosis; and (c) patients unable to answer the questionnaire. The study protocol was approved by the Ethics Committee of the Fujian Medical University, and written informed consent was obtained from all patients prior to their enrolment (Code: [2014] (98)).

### Collection of baseline information

A structured questionnaire was designed for this study. Data was collected during face-to-face interviews with patients conducted by trained investigators. Data pertaining to the following variables were collected: general condition (age, sex, education level, height, and weight), history of smoking and alcohol consumption, physical activity, and baseline quality of life (QoL) scores. This data was collected at the time of admission to the hospital.

The International Physical Activity Questionnaire long form (IPAQ-L) was used to assess the level of physical activity [23, 24]. The IPAQ-L covers four domains (work or study, transportation, household duties, and sports leisure) and explores physical activity during the seven days immediately preceding the date of admission to the hospital. The number of days and the number of minutes in a day spent on physical activities were listed in the IPAQ-L. Data cleaning was undertaken to exclude any missing activity frequency or time data as well as any self-reported total time of physical activity of more than 960 min (the study assumed that each person had at least eight hours of sleep). Activity corresponding to time and weekly frequency was re-coded as 0 if the total time of physical activity was less than 10 min a day because at least 10 min of continuous physical activity could lead to good health outcomes). We then used the secondary truncation rule to calculate the level of physical activity. Firstly, if the daily duration of physical activity of a certain intensity exceeded 3 h, it was re-coded as 180 min. This principle allows for a maximum of 21 h (1260 min) per week of reported physical activity of each intensity level. Then, based on the rule of first truncation, the cumulative weekly hours of activities of the same intensity were added up and re-coded as 1260 min if the total time exceeded 1260 min. The physical activity level (MET-min/w) was calculated every week by the MET assigned to the physical activity (Supplement Table 1) multiplied by the weekly frequency (d/w) and the time spent each day (min/d). The sum of different intense physical activity levels was the total physical activity level. According to the IPAQ Working Group (Supplement Table 2), we re-coded the physical activity level (MET-min/w) into three gradient levels (low-level, moderate-level, and high-level).

**Table 1:** Characteristics of study patients in demographics and clinical message at baseline

Characteristic	*n* (%)	Levels of physical activity (*n*=376)			χ^2^	*P*
		Low-level *N*=44 *n*(%)	Moderate-level *N*=118 *n*(%)	High-level *N*=214 *n*(%)		
Age					5.260	0.072
≤60	190 (50.5)	29 (65.9)	54 (45.8)	107 (50.0)		
>60	186 (49.5)	15 (34.1)	64 (54.2)	107 (50.0)		
Gender					30.837	**<0.001**
Male	162 (43.1)	28 (63.6)	68 (57.6)	66 (30.8)		
Female	214 (56.9)	16 (36.4)	50 (42.4)	148 (69.2)		
BMI					6.729	0.151
<18.5	22 (6.0)	3 (6.8)	9 (7.8)	10 (4.9)		
[18.5, 24)	217 (59.6)	24 (54.5)	77 (67.0)	116 (56.6)		
≥24	125 (34.3)	17 (38.6)	29 (25.2)	79 (38.5)		
Marital status					2.851	0.240
Single (included divorced or widowed)	34 (9.1)	1 (2.3)	12 (10.3)	21 (9.9)		
In a relationship	338 (90.9)	43 (97.7)	104 (89.7)	191 (90.1)		
Family income per month					0.731	0.694
≤10000	208 (57.9)	26 (61.9)	61 (55.0)	121 (58.7)		
>10000	151 (42.1)	16 (38.1)	50 (45.0)	85 (41.3)		
Educational level					8.587	**0.014**
Primary and below	201 (54.0)	19 (44.2)	53 (45.7)	129 (60.6)		
Junior high school and above	171 (46.0)	24 (55.8)	63 (54.3)	84 (39.4)		
Smoker					10.895	**0.004**
No	258 (68.6)	24 (54.5)	73 (61.9)	161 (75.2)		
Yes	118 (31.4)	20 (45.5)	45 (38.1)	53 (24.8)		
Drinker					9.818	**0.007**
No	297 (79.8)	30 (68.2)	87 (74.4)	180 (85.3)		
Yes	75 (20.2)	14 (31.8)	30 (25.6)	31 (14.7)		
TNM stage					2.367	0.306
0 and I	167 (73.9)	14 (60.9)	45 (73.8)	108 (76.1)		
II and above	59 (26.1)	9 (39.1)	16 (26.2)	34 (23.9)		
Maximum diameter of tumor					0.144	0.930
≤2.0	227 (63.9)	23 (62.2)	70 (63.1)	134 (64.7)		
>2.0	128 (36.1)	14 (37.8)	41 (36.9)	73 (35.3)		
Therapeutic method					5.071	0.535
Untreated	15 (4.0)	2 (4.5)	4 (3.4)	9 (4.2)		
Surgery alone	275 (73.1)	28 (63.6)	84 (71.2)	163 (76.2)		
Chemotherapy/radiation alone	17 (4.5)	4 (9.1)	6 (5.1)	7 (3.3)		
Treated with both chemotherapy/radiation and surgery	69 (18.4)	10 (22.7)	24 (20.3)	35 (16.4)

**Table 2:** Baseline of patients QoL scores

	Levels of physical activity (*n*=376)			*H*	*P*
	Low-level (M(P_25_,P_75_))	Moderate-level (M(P_25_,P_75_))	High-level (M(P_25_,P_75_))		
QLQ-C30					
Global health status (QL)	75.00 (66.67, 83.33)	75.00 (66.67, 83.33)	83.33 (66.67, 83.33)	12.423	**0.002**
* Functional scales*					
Physical functioning (PF)	96.67 (81.67, 100.00)	93.33 (86.67, 100.00)	93.33 (86.67, 100.00)	0.086	0.958
Role functioning (RF)	100.00 (66.67, 100.00)	100.00 (100.00, 100.00)	100.00 (100.00, 100.00)	3.808	0.149
Emotional functioning (EF)	91.67 (83.33, 100.00)	83.33 (75.00, 100.00)	83.33 (75.00, 100.00)	2.841	0.242
Cognitive functioning (CF)	100.00 (100.00, 100.00)	100.00 (83.33, 100.00)	100.00 (83.33, 100.00)	0.724	0.696
Social functioning (SF)	83.33 (66.67, 100.00)	66.67 (66.67, 100.00)	66.67 (66.67, 100.00)	0.792	0.673
* Symptom scales/items*					
Fatigue (FA)	11.11 (0.00, 22.22)	11.11 (0.00, 33.33)	11.11 (0.00, 33.33)	0.311	0.856
Nausea and vomiting (NV)	0.00 (0.00, 0.00)	0.00 (0.00, 0.00)	0.00 (0.00, 0.00)	2.163	0.339
Pain (PA)	8.33 (0.00, 33.33)	0.00 (0.00, 16.67)	0.00 (0.00, 16.67)	1.920	0.383
Dyspnea (DY)	0.00 (0.00, 33.33)	0.00 (0.00, 33.33)	0.00 (0.00, 33.33)	2.060	0.357
Insomnia (SL)	0.00 (0.00, 33.33)	0.00 (0.00, 33.33)	0.00 (0.00, 33.33)	3.757	0.153
Appetite loss (AP)	0.00 (0.00, 0.00)	0.00 (0.00, 0.00)	0.00 (0.00, 33.33)	2.082	0.353
Constipation (CO)	0.00 (0.00, 0.00)	0.00 (0.00, 0.00)	0.00 (0.00, 0.00)	0.228	0.892
Diarrhea (DI)	0.00 (0.00, 0.00)	0.00 (0.00, 0.00)	0.00 (0.00, 0.00)	0.281	0.869
Financial difficulties (FI)	0.00 (0.00, 33.33)	0.00 (0.00, 33.33)	0.00 (0.00, 33.33)	3.310	0.191
QLQ-LC13					
Dyspnea (LC-DY)	0.00 (0.00, 11.11)	0.00 (0.00, 11.11)	0.00 (0.00, 11.11)	0.666	0.717
Coughing (LC-CO)	16.67 (0.00, 33.33)	33.33 (0.00, 33.33)	0.00 (0.00, 33.33)	2.695	0.260
Hemoptysis (LC-HA)	0.00 (0.00, 0.00)	0.00 (0.00, 0.00)	0.00 (0.00, 0.00)	1.262	0.532
Sore mouth (LC-SM)	0.00 (0.00, 0.00)	0.00 (0.00, 0.00)	0.00 (0.00, 0.00)	0.651	0.722
Dysphagia (LC-DS)	0.00 (0.00, 0.00)	0.00 (0.00, 0.00)	0.00 (0.00, 0.00)	0.717	0.699
Peripheral neuropathy (LC-PN)	0.00 (0.00, 0.00)	0.00 (0.00, 0.00)	0.00 (0.00, 0.00)	0.457	0.796
Alopecia (LC-HR)	0.00 (0.00, 0.00)	0.00 (0.00, 0.00)	0.00 (0.00, 0.00)	1.907	0.385
Pain in chest (LC-PC)	0.00 (0.00, 33.33)	0.00 (0.00, 0.00)	0.00 (0.00, 33.33)	3.258	0.196
Pain in aim or should (LC-PA)	0.00 (0.00, 0.00)	0.00 (0.00, 0.00)	0.00 (0.00, 0.00)	3.073	0.215
Pain in other parts (LC-PO)	0.00 (0.00, 0.00)	0.00 (0.00, 0.00)	0.00 (0.00, 0.00)	2.167	0.338

### Quality of life assessments

The European Organisation for Research and Treatment of Cancer (EORTC) Quality of Life Questionnaire version 3.0 (EORTC QLQ-C30) and EORTC Quality of Life Questionnaire-Lung Cancer (EORTC QLQ-LC13) were used to assess the quality of life of patients at baseline and during follow-up. EORTC QLQ-C30 is a 30-item generic questionnaire that includes five functioning scales, three symptom scales, and a global health scale [25]. The EORTC QLQ-LC13 module comprises 13 questions for the assessment of lung cancer-associated symptoms, treatment-related side effects, and use of pain medication [26] (Supplement Table 3). For both the EORTC QLQ-C30 and QLQ-LC13, raw scores are transformed into scale scores ranging from 0 to 100. Higher scores reflect better HRQoL on the global health scale and functioning scales of QLQ-C30, while high scores are related to a high symptomatic level in symptom scales in QLQ-C30 and all scales in QLQ-LC13.

**Table 3:** Comparison of time to deterioration in different level of physical activity and univariate cox regression analysis of physical activity level and time to deterioration event ≥ 5 points

	Time to deterioration event *n*(%)			χ^2^	*P*	Time to deterioration M (P_25_, P_75_)			*HR* (95% *CI*)	*P*
	Low-level	Moderate-level	High-level			Low-level	Moderate-level	High-level		
QLQ-C30										
Global health status (QL)	22 (50.0)	40 (33.9)	92 (43.0)	4.285	0.117	21.70 (10.82, 33.18)	20.80 (13.59, 27.77)	22.14 (12.98, 29.13)	1.035 (0.826-1.298)	0.762
* Functional scales*										
Physical functioning (PF)	23 (52.3)	58 (49.2)	96 (44.9)	1.103	0.576	22.19 (10.99, 29.23)	19.86 (12.61, 25.91)	21.39 (12.53, 29.81)	0.902 (0.736-1.105)	0.319
Role functioning (RF)	15 (34.1)	38 (32.2)	63 (29.4)	0.518	0.772	25.43 (18.79, 34.18)	20.80 (13.22, 26.72)	22.77 (12.90, 32.44)	0.910 (0.706-1.173)	0.467
Emotional functioning (EF)	11 (25.0)	22 (18.6)	35 (16.4)	1.877	0.391	25.79 (19.37, 34.98)	22.97 (14.13, 30.87)	23.85 (13.42, 32.89)	0.821 (0.594-1.134)	0.232
Cognitive functioning (CF)	13 (29.5)	29 (24.6)	47 (22.0)	1.240	0.538	25.43 (18.79, 34.34)	22.90 (14.02, 29.24)	23.85 (13.26, 31.96)	0.877 (0.661-1.165)	0.366
Social functioning (SF)	13 (29.5)	29 (24.6)	39 (18.2)	3.704	0.157	26.20 (19.66, 34.98)	22.75 (13.32, 29.96)	23.84 (13.71, 32.89)	0.819 (0.611-1.096)	0.179
* Symptom scales/items*										
Fatigue (FA)	15 (34.1)	32 (27.1)	49 (22.9)	2.633	0.268	25.79 (19.37, 34.18)	22.31 (13.12, 26.49)	23.00 (13.03, 31.64)	0.845 (0.645-1.107)	0.221
Nausea and vomiting (NV)	9 (20.5)	12 (10.2)	27 (12.6)	3.054	0.217	26.20 (19.79, 35.33)	23.03 (14.13, 31.75)	23.89 (13.36, 32.99)	0.879 (0.598-1.294)	0.514
Pain (PA)	15 (34.1)	36 (30.5)	56 (26.2)	1.481	0.477	25.38 (18.79, 34.18)	20.19 (13.17, 26.32)	22.49 (12.53, 31.44)	0.908 (0.702-1.174)	0.462
Dyspnea (DY)	22 (50.0)	37 (31.4)	62 (29.0)	7.448	**0.024**	25.79 (19.37, 34.18)	22.75 (13.84, 29.24)	23.08 (12.90, 31.84)	0.827 (0.652-1.049)	0.117
Insomnia (SL)	18 (40.9)	33 (28.0)	48 (22.4)	6.662	**0.036**	25.43 (13.32, 33.72)	22.55 (13.26, 29.24)	23.36 (13.15, 32.18)	0.759 (0.586-0.982)	**0.036**
Appetite loss (AP)	15 (34.1)	23 (19.5)	39 (18.2)	5.745	0.057	25.79 (19.37, 35.33)	22.90 (13.84, 29.89)	23.84 (13.32, 32.74)	0.788 (0.588-1.057)	0.111
Constipation (CO)	10 (22.7)	22 (18.6)	28 (13.1)	3.456	0.178	25.79 (19.37, 34.80)	22.97 (14.07, 31.75)	23.95 (13.49, 33.48)	0.751 (0.538-1.049)	0.093
Diarrhea (DI)	13 (29.5)	20 (16.9)	29 (13.6)	6.806	**0.033**	23.74 (18.37, 33.18)	23.03 (14.07, 31.75)	24.00 (13.71, 33.07)	0.677 (0.489-0.938)	**0.019**
Financial difficulties (FI)	12 (27.3)	30 (25.4)	47 (22.0)	0.862	0.650	25.38 (18.79, 33.72)	22.90 (13.64, 29.96)	23.85 (13.61, 32.91)	0.865 (0.645-1.161)	0.334
QLQ-LC13										
Dyspnea (LC-DY)	25 (56.8)	54 (45.8)	83 (38.8)	5.343	0.069	20.11 (9.24, 29.23)	19.86 (12.58, 25.91)	21.95 (12.22, 30.09)	0.798 (0.647-0.985)	**0.036**
Coughing (LC-CO)	15 (34.1)	36 (30.5)	71 (33.2)	0.309	0.857	25.61 (19.37, 34.98)	21.50 (13.27, 27.77)	22.08 (12.53, 30.40)	1.085 (0.842-1.400)	0.528
Hemoptysis (LC-HA)	7 (15.9)	11 (9.3)	19 (8.9)	2.085	0.353	26.20 (19.79, 35.63)	23.18 (14.20, 33.19)	24.72 (13.91, 34.05)	0.807 (0.526-1.238)	0.326
Sore mouth (LC-SM)	11 (25.0)	10 (8.5)	18 (8.4)	11.470	**0.003**	26.50 (19.66, 35.33)	23.18 (14.37, 33.19)	24.72 (13.93, 34.07)	0.632 (0.425-0.940)	**0.024**
Dysphagia (LC-DS)	8 (18.2)	15 (12.7)	17 (7.9)	4.802	0.091	26.20 (19.66, 34.98)	22.90 (14.07, 30.87)	24.82 (13.93, 33.98)	0.658 (0.443-0.978)	**0.038**
Peripheral neuropathy (LC-PN)	11 (25.0)	21 (17.8)	37 (17.3)	1.483	0.476	26.20 (19.66, 34.98)	22.90 (14.20, 32.78)	24.57 (13.93, 33.48)	0.836 (0.606-1.154)	0.276
Alopecia (LC-HR)	9 (20.5)	17 (14.4)	28 (13.1)	1.612	0.447	25.79 (19.37, 34.18)	23.11 (14.20, 32.78)	23.98 (13.36, 33.07)	0.820 (0.571-1.177)	0.282
Pain in chest (LC-PC)	13 (29.5)	30 (25.4)	48 (22.4)	1.147	0.563	22.19 (15.14, 33.72)	20.52 (13.12, 26.32)	23.08 (13.07, 32.49)	0.868 (0.658-1.146)	0.318
Pain in aim or should (LC-PA)	9 (20.5)	22 (18.6)	29 (13.6)	2.222	0.329	26.20 (19.66, 34.98)	22.90 (14.07, 29.24)	23.85 (13.51, 32.89)	0.812 (0.582-1.134)	0.221
Pain in other parts (LC-PO)	15 (34.1)	34 (28.8)	57 (26.6)	1.035	0.596	25.61 (19.37, 35.33)	20.31 (13.59, 28.63)	23.44 (13.26, 32.44)	0.931 (0.720-1.204)	0.585

### Follow-up

Survival time was defined as time from surgery (May 31, 2017–December 6, 2020) to death or the end of follow-up on September 11, 2021. All patients were followed up every 3–6 months in the first year, and annually thereafter.

### Time to deterioration model

TTD was defined as the time from inclusion in the study to the first clinically meaningful deterioration compared to the baseline HRQoL scores in the respective HRQoL assessment tools [27]. The minimal clinically important difference refers to the smallest difference in HRQoL scores perceived as clinically important; it is an important indicator for judging the clinical relevance of the results [28]. In our study, TTD was defined as the time from the first observation with definitive deterioration with a > 5-point, and no subsequent observations with a <5-point decrease compared to baseline in the EORTC QLQ-C30 and EORTC QLQ-LC13 [29].

### Statistical analysis

The QoLR package was used to calculate the HRQoL scores and determine the TTD events in EORTC QLQ-C30 and EORTC QLQ-LC13. Median and interquartile range were used to describe the HRQoL scores and TTD. And chi-squared test was performed to assess the differences in sociodemographic, clinical characteristics, and incidence rate of TTD events between patients with different levels of physical activity. Baseline HRQoL scores of three physical activity levels were compared using the Kruskal-Wallis test. Survival analysis was performed using the univariate and multiple Cox regression analysis after controlling for confounding factors; the results are shown as hazard ratios (HRs) with 95% confidence intervals (CIs). All statistical analyses were performed using R software (version 3.5.2) and Statistical Product and Service Solutions version 20.0 (SPSS 20.0).

## Results

### Sociodemographic, clinical characteristics, and HRQoL scores at baseline

A total of 440 participants completed the baseline questionnaire with a pathological diagnosis of primary LUAD. Among the 440 LUAD patients, 376 LUAD patients completed the first time EORTC QLQ-C30 and QLQ-LC13, 147 patients completed the second time, 80 patients completed the third time, 21 patients completed the fourth time, and three patients completed the fifth time. All patients included in our analysis (*n*=376) completed the baseline questionnaire and at least one follow-up EORTC QLQ-C30 and QLQ-LC13. Twenty-five patients died during the follow-up period and the median follow-up time was 25 months [19, 30]. Sixty-four patients dropped out during the follow-up (drop-out rate: 17.0%).

The sociodemographic and clinical characteristics of LUAD patients with different physical activity levels are shown in Table 1. There were significant differences between the three levels of physical activity with respect to the distribution of sex, education level, and history of smoking and alcohol consumption (*P*<0.05). However, there were no significant between-group differences with respect to age, body mass index (BMI), marital status, income, TNM stage, maximum tumor diameter, or therapeutic method. HRQoL scores are presented as a median and interquartile range in Table 2. Only QL scale scores showed significance differences between the three levels of physical activity.

### Time to deterioration and HRQoL events

**Fig. 1:**
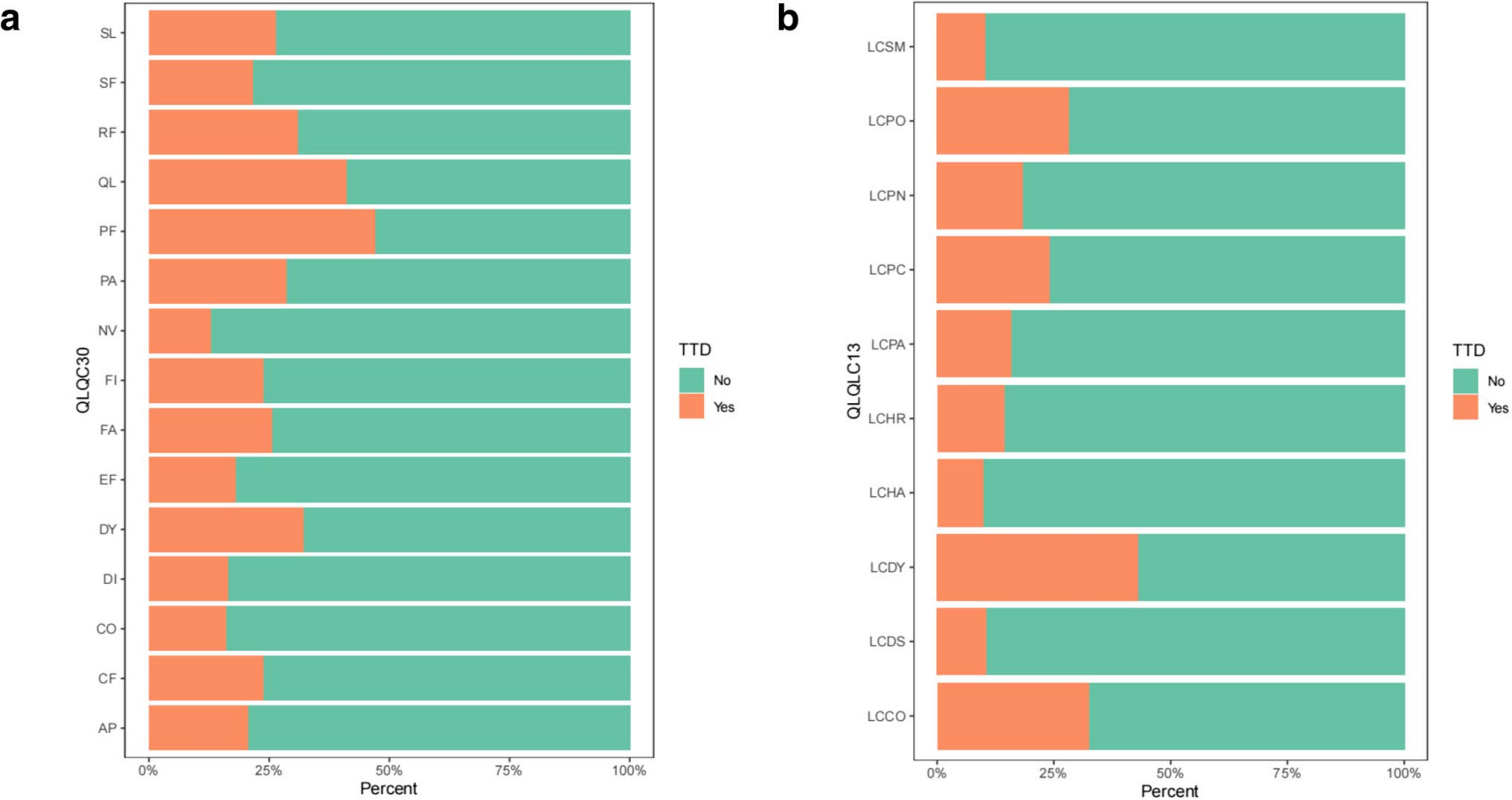
The occurrence of TTD events in EORTC QLQ-C30 (**a**) and EORTC QLQ-LC13 (**b**)

**Fig. 2:**
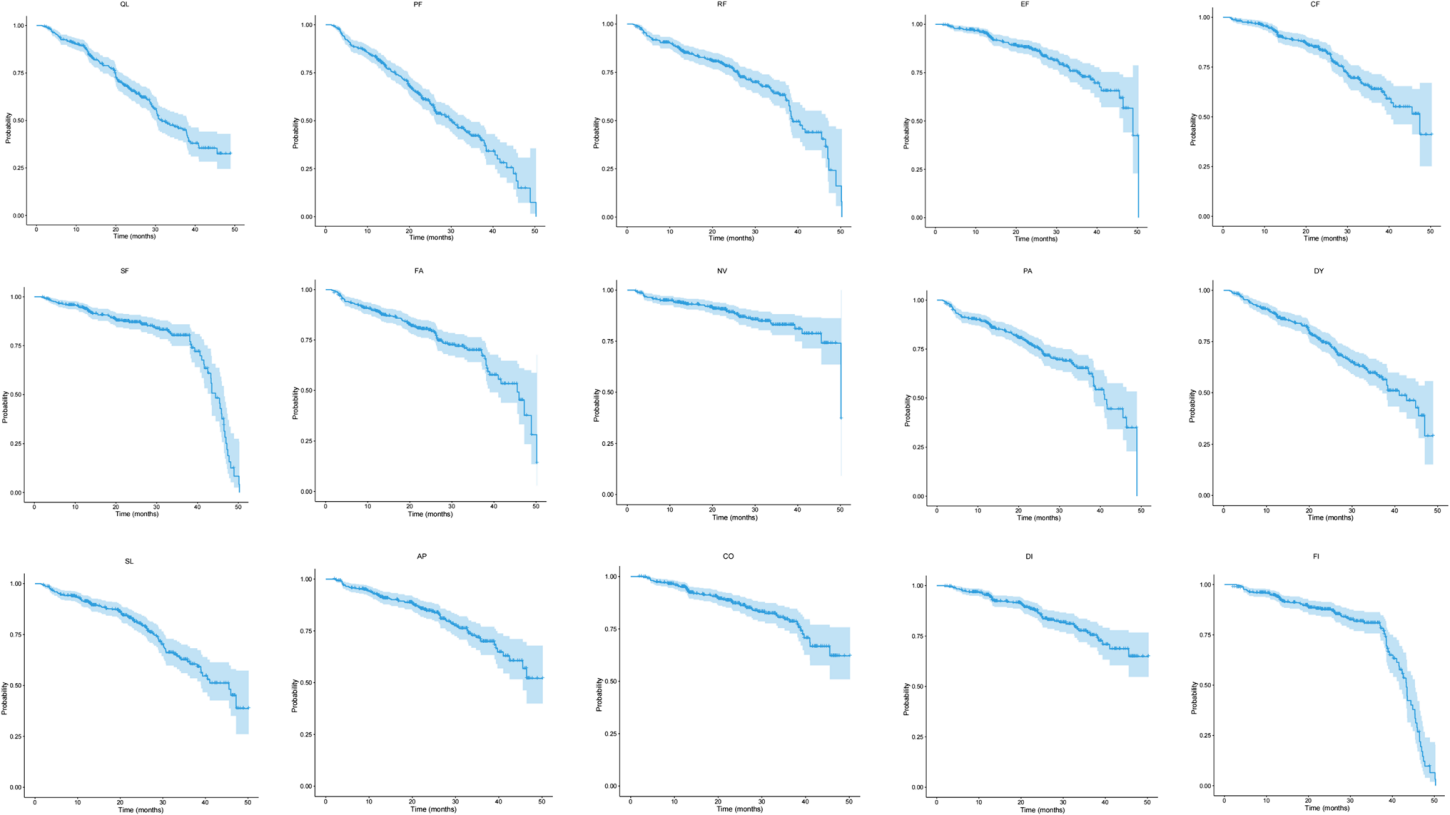
The TTD of all EORTC QLQ-C30 scales

**Fig. 3:**
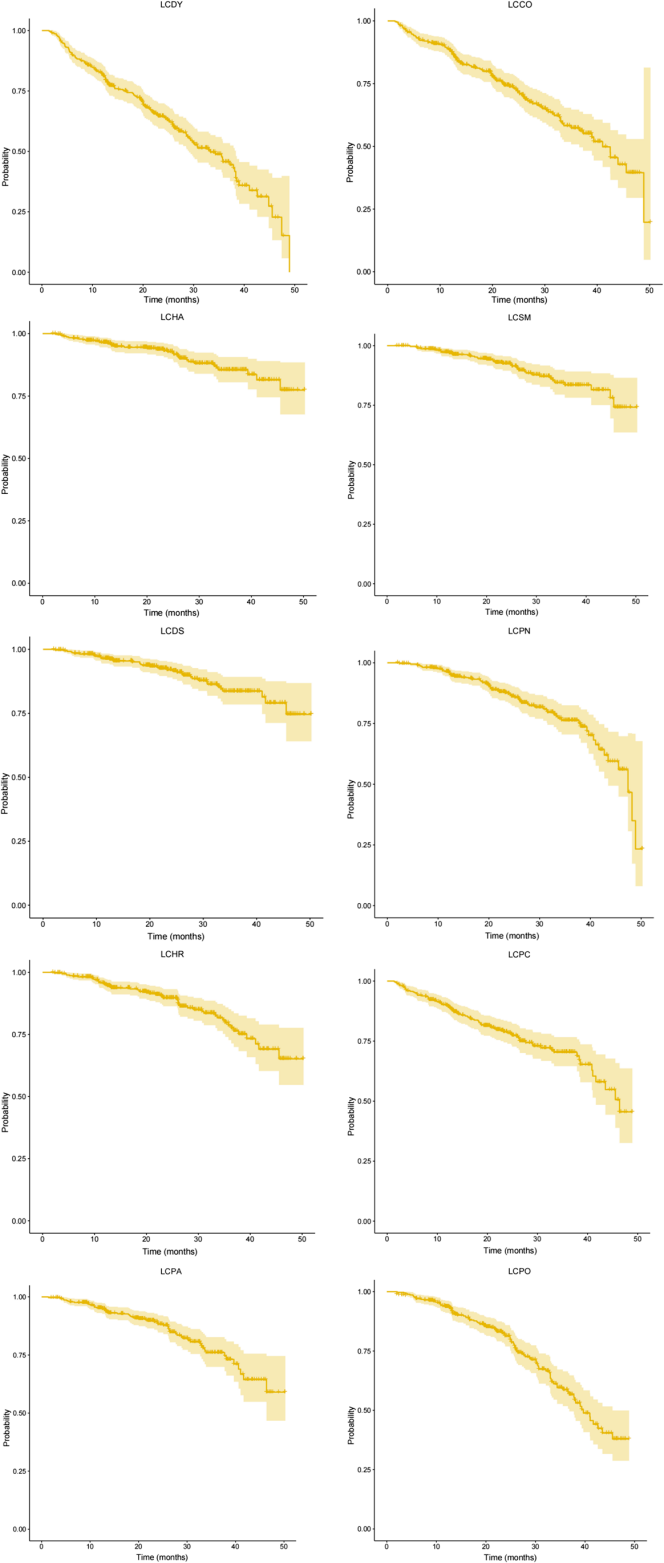
The TTD of all EORTC QLQ-LC13 scales

In the functioning scales of EORTC QLQ-C30, time to physical functioning (PF) deterioration event was the most common in our cohort during follow-up, while dyspnea (DY) was the most common in symptom scales of QLQ-C30 (Fig. 1a). The occurrence of TTD of dyspnea (LC-DY) events in EORTC QLQ-LC13 was the first, and coughing (LC-CO) was the second (Fig. 1b). TTD was calculated using the Kaplan-Meier method, HRQoL decreased over time. TTD in all scales of EORTC QLQ-C30 and LC13 are shown in Figs. 2 and 3.

### Association between TTD and physical activity

As shown in Table 3, the low-level physical activity group had a significantly higher proportion of patients with deterioration dyspnea (DY) events (*P*=0.024), insomnia (SL) events (*P*=0.036), and diarrhea (DI) events (*P*=0.033) in EORTC QLQ-C30. Deterioration of sore mouth (LC-SM) (*P*=0.003) was also higher in the low-level physical activity group. Three levels of physical activity were used as continuous variables to explore the association of physical activity with the HRQoL of LUAD patients. On univariate Cox regression analysis, a higher level of physical activity was associated with improved HRQoL of insomnia (SL) (*HR*=0.759, 95% *CI*: 0.586–0.982, *P*=0.036), diarrhea (DI) (*HR*=0.677, 95% *CI*: 0.489–0.938, *P*=0.019), dyspnea (LC-DY) (*HR*=0.798, 95% *CI*: 0.647–0.985, *P*=0.036), sore mouth (LC-SM) (*HR*=0.632, 95% *CI*: 0.425–0.940, *P*=0.029), and dysphagia (LC-DS) (*HR*=0.658, 95% *CI*: 0.443–0.978, *P*=0.038).

To minimize the influence of potential confounding factors, we adjusted for all baseline variables (including age, sex, education level, BMI, history of smoking, and alcohol consumption) and clinical variables (including stage, maximum tumor diameter, therapeutic method) in the multiple Cox regression analysis. The results obtained were similar to the univariate analysis. Physical activity was associated with reduced incidence of time to deterioration in insomnia (SL) (*HR*=0.635, 95%*CI*: 0.437–0.922, *P*=0.017), diarrhea (DI) (*HR*=0.475, 95%*CI*: 0.291–0.774, *P*=0.003), dyspnea (LC-DY) (*HR*=0.654, 95%*CI*: 0.474–0.903, *P*=0.010), sore mouth (LC-SM) (*HR*=0.457, 95%*CI*: 0.244–0.856, *P*=0.015), and dysphagia (LC-DS) (*HR*=0.315, 95%*CI*: 0.172–0.580, *P*<0.001) (Table 4).

**Table 4:** Multivariate Cox analysis for time to deterioration event ≥ 5 points

Items	*HR* (95%*CI*)	*P*
QLQ-C30		
* Insomnia (SL)*	0.635 (0.437-0.922)	**0.017**
* Diarrhea (DI)*	0.475 (0.291-0.774)	**0.003**
QLQ-LC-13		
* Dyspnea (LC-DY)*	0.654 (0.474-0.903)	**0.010**
* Sore mouth (LC-SM)*	0.457 (0.244-0.856)	**0.015**
* Dysphagia (LC-DS)*	0.315 (0.172-0.580)	**<0.001**

Adjusted for all baseline variables (including age, gender, marital status, income, education, BMI, smoking and drinking) and clinical variables (including stage, maximum diameter of tumor, therapeutic method), boldface means *P*<0.05

## Discussion

With the improvement in survival time of LUAD patients, HRQoL is increasingly being recognized as a key factor impinging on the prognosis of these patients. In this study, we constructed a TTD model for LUAD including EORTC QLQ-C30 and QLQ-LC13 in a prospective study. We identified that pre-treatment physical activity levels affected the TTD of insomnia, diarrhea, dyspnea, sore mouth, and dysphagia.

EORTC QLQ-C30 is widely used to assess HRQoL in the context of many cancers [31]. A previous study found a significant decrease in the EORTC QLQ-C30 score and a decrease in HRQoL in social, physical, and role functioning and in the dyspnea symptom score after therapy [32]. Similar results were found in our study, in that all scales in EORTC QLQ-C30 and LC-13 decreased over time. In functioning scales, TTD events of physical functioning were the most common, while role functioning was the second most common. TTD of dyspnea was also the first in symptom scales, in both QLQ-C30 and QLQ-LC13 (DY and LC-DY). These findings indicated that physical and role functioning and dyspnea symptoms warrant more clinical attention. Pre-surgery exercise has been shown to have substantially beneficial effects on lung cancer [33]. Our assessment of the association between TTD of HRQoL and physical activity also revealed that higher-level physical activity before treatment can significantly delay the TTD of insomnia (SL), diarrhea (DI), dyspnea (LC-DY), sore mouth (LC-SM), and dysphagia (LC-DS).

Insomnia is one of the most common sleep disorders, which seriously affects the daily life of patients [34]. Physical activity has been shown to reduce the incidence of insomnia, and insomnia is less prevalent in physically active individuals compared to individuals with a sedentary lifestyle [30, 35–37]. Increased daily physical activity of patients with cancer has been shown to improve sleep and alleviate insomnia [38, 39]. Physical inactivity has also been shown to be associated with gastrointestinal symptoms [40, 41]. A randomized controlled trial investigated the impact of exercise on the HRQoL of patients with prostate cancer and found that patients with exercise intervention had fewer diarrhea symptoms [42]. Another study on prostate cancer survivors also obtained the same results, with physical activity associated with an improvement in diarrhea [43]. Similar results were found in our study, suggesting that physical activity can significantly delay the deterioration of insomnia and diarrhea.

Dyspnea is one of the most common symptoms in patients with lung cancer. Increased physical activity over time has been shown to improve dyspnea after thoracic radiation therapy in patients with breast cancer, lung cancer, and lymphoma [44]. Another study conducted in Korea also observed an association of moderate to vigorous physical activity with fewer symptoms of dyspnea among breast and colorectal cancer survivors [45]. TTD of dyspnea (DY) in QLQ-C30 did not seem to be related to physical activity in our study; however, deterioration of dyspnea (LC-DY) measured by QLQ-LC13 was significantly delayed by physical activity. Sore mouth is a prominent symptom in cancer patients receiving chemotherapy [46, 47]. In patients with non-small cell lung cancer, chemotherapy with cisplatin or anlotinib was found to aggravate sore mouth [48, 49]. However, physical activity was found to delay the exacerbation of sore mouth in the current study, suggesting that physical activity may be applied as a non-pharmaceutical intervention to improve sore mouth in LUAD patients undergoing chemotherapy. Dysphagia is a persistent symptom in cancer patients after mediastinal radiation and chemotherapy [50] and has been shown to be associated with a worse prognosis and HRQoL in lung cancer patients [51, 52]. Methods to improve dysphagia are an important area of lung cancer therapy research. Higher levels of pre-treatment physical activity were found to significantly slow down the TTD of dysphagia in our prospective study. Exercise is an approach to improve muscular coordination and reduce pain in the masticatory muscles (53); dysphagia and sore mouth were associated with muscles and may also be reduced by exercise. The above results suggest that lifestyle interventions to improve physical activity may improve the HRQoL of patients with lung cancer.

To the best of our knowledge, this is the first prospective study to explore the relationship between HRQoL and physical activity based on a TTD model analysis. Our findings may provide a new perspective to improve the quality of life for patients with LUAD. Nevertheless, some limitations of our study should be acknowledged. Post-operative exercise also has a positive impact on the prognosis of many cancers; however, in this study, we did not assess the effect of post-treatment physical activity on the HRQoL. Second, 64 patients withdrew from our study probably due to disease progression or deterioration over a short time after therapy, or due to lack of follow-up. Thus, it is inevitable that there was some follow-up bias in our study causing biased exposure-outcome association estimates. Lastly, the IPAQ scale reflects the physical activity in the preceding seven days; however, physical activity levels 7 days prior to admission are likely to be affected by illness. The self-reported IPAQ scale also contained recall bias.

## Conclusions

Pre-treatment physical activity is a modifiable factor that can delay the TTD of insomnia, diarrhea, dyspnea, sore mouth, and dysphagia, as assessed by EORTC QLQ-C30 and EORTC QLQ-LC13. Our report allowed us to generate the hypothesis that pre-treatment physical activity may help maintain a stable HRQoL in patients with LUAD.

### Supplementary Information

Below is the link to the electronic supplementary material.

Supplementary file1 (82.0 KB)
