# Supplementary material for: Association between physical activity and health-related quality of life: time to deterioration model analysis in lung adenocarcinoma
Source: J Cancer Surviv. 2022 Oct 4;17(6):1769–79. doi: 10.1007/s11764-022-01259-z (PMC10539423; doi:10.1007/s11764-022-01259-z)
Supplement: Supplementary file 1 — Supplementary file1 (82.0 KB) [file 11764_2022_1259_MOESM1_ESM.doc]

| Supplemently Table 1 Physical activity attributes and MET values in IPAQ | | | |
| --- | --- | --- | --- |
| Type | Item | Strengh of physical activity | MET value |
| Work-related | Walk | Walk | 3.3 |
|  | Moderate-strength | Moderate | 4.0 |
|  | High-strength | High | 8.0 |
| Transportation-related | Walk | Walk | 3.3 |
|  | Ride | Moderate | 6.0 |
| Household-related | Moderate-streng outdoor housework | Moderate | 3.0 |
|  | Moderate-strength indoor housework | Moderate | 4.0 |
|  | High-strength outdoor housework | Moderate | 5.5 |
| Leisure-related | Walk | Walk | 3.3 |
|  | Moderate-strength | Moderate | 4.0 |
|  | High-strength | High | 8.0 |

| Supplemently Table 2 Standardize of physical activity level | |
| --- | --- |
| Physical activity level | Standardization |
| High-level | Meet any of the following 2 criteria: |
|  | 1. All kinds of high-strength physical activity were carried out ≥ 3 days, and the weekly total physical activity level ≥1 500 met-min /w |
|  | 2. The three levels of physical activity totaled ≥ 7 days, and the total weekly physical activity level ≥ 3 000 met-min /w |
| Moderate-level | Meet any of the following 3 criteria: |
|  | 1. All kinds of high-strength physical activity for at least 20 minutes per day≥3 days |
|  | 2. At least 30 minutes of moderate-strength and/or walking activity per day ≥ 5 days |
|  | 3. The three levels of physical activity were carried out ≥ 5 days, and the total weekly physical activity level ≥ 600 met-min /w |
| Low-level | Meet any of the following 2 criteria: |
|  | 1. No physical activity was reported |
|  | 2. Some activities were reported but did not meet the above standardization of moderate or high-level |

| Supplemently Table 3 Scales of EORTC QLQ-C30 and EORTC QLQ-LC13 | | | |
| --- | --- | --- | --- |
| **EORTC QLQ-C30** | No. items | Degrees of freedom | Item number |
| Global health status (QL) | 2 | 6 | 29,30 |
| ***Functional scales*** |  |  |  |
| Physical functioning (PF) | 5 | 3 | 1-5 |
| Role functioning (RF) | 2 | 3 | 6,7 |
| Emotional functioning (EF) | 4 | 3 | 21-24 |
| Cognitive functioning (CF) | 2 | 3 | 20,25 |
| Social functioning (SF) | 2 | 3 | 26,27 |
| ***Symptom scales/items*** |  |  |  |
| Fatigue (FA) | 3 | 3 | 10,12,18 |
| Nausea and vomiting (NV) | 2 | 3 | 14,15 |
| Pain (PA) | 2 | 3 | 9,19 |
| Dyspnoea (DY) | 1 | 3 | 8 |
| Insomnia (SL) | 1 | 3 | 11 |
| Appetite loss (AP) | 1 | 3 | 13 |
| Constipation (CO) | 1 | 3 | 16 |
| Diarrhoea (DI) | 1 | 3 | 17 |
| Financial difficulties (FI) | 1 | 3 | 28 |
|  |  |  |  |
| **QLQ-LC13** | No. items | Degrees of freedom | Item number |
| Dyspnoea (LC-DY) | 3 | 3 | 33,34,35 |
| Coughing (LC-CO) | 1 | 3 | 31 |
| Haemoptysis (LC-HA) | 1 | 3 | 32 |
| Sore mouth (LC-SM) | 1 | 3 | 36 |
| Dysphagia (LC-DS) | 1 | 3 | 37 |
| Peripheral neuropathy (LC-PN) | 1 | 3 | 38 |
| Alopecia (LC-HR) | 1 | 3 | 39 |
| Pain in chest (LC-PC) | 1 | 3 | 40 |
| Pain in aim or should (LC-PA) | 1 | 3 | 41 |
| Pain in other parts (LC-PO) | 1 | 3 | 42 |
